# Field Effectiveness of a Typhoid Conjugate Vaccine: The 2018 Navi Mumbai Pediatric TCV Campaign

**DOI:** 10.4269/ajtmh.24-0181

**Published:** 2024-08-13

**Authors:** Kashmira Date, Christopher LeBoa, Seth A. Hoffman, Pradeep Haldar, Pauline Harvey, Qian An, Chenhua Zhang, Vijay N. Yewale, Savita Daruwalla, Dhanya Dharmapalan, Jeetendra Gavhane, Shrikrishna Joshi, Rajesh Rai, Varsha Rathod, Keertana Shetty, Divyalatha S. Warrier, Shalini Yadav, Rahul Shimpi, Niniya Jayaprasad, Lily Horng, Kirsten Fagerli, Priyanka Borhade, Debjit Chakraborty, Arun Katkar, Abhishek Kunwar, Jason R. Andrews, Sunil Bahl, Pankaj Bhatnagar, Shanta Dutta, Stephen P. Luby

**Affiliations:** ^1^ Global Immunization Division, Center for Global Health, Centers for Disease Control and Prevention, Atlanta, Georgia;; ^2^ Division of Infectious Diseases and Geographic Medicine, Department of Medicine, Stanford University School of Medicine, Stanford, California;; ^3^ Ministry of Health & Family Welfare, Government of India, New Delhi, India;; ^4^ World Health Organization-Country Office for India, National Public Health Surveillance Project, New Delhi, India;; ^5^ Dr. Yewale Multispecialty Hospital for Children, Navi Mumbai, India;; ^6^ Department of Pediatrics, NMMC General Hospital, Navi Mumbai, India;; ^7^ Department of Pediatrics, MGM New Bombay Hospital, MGM Medical College, Navi Mumbai, India;; ^8^ Dr. Joshi’s Central Clinical Microbiology Laboratory, Navi Mumbai, India;; ^9^ Department of Pediatrics & Neonatology, Dr. D.Y. Patil Medical College and Hospital, Navi Mumbai, India;; ^10^ Rajmata Jijau Hospital, Airoli (NMMC), Navi Mumbai, India;; ^11^ Department of Microbiology, Dr. D.Y. Patil Medical College and Hospital, Navi Mumbai, India;; ^12^ Department of Pediatrics, Mathadi Trust Hospital, Navi Mumbai, India;; ^13^ Department of Microbiology, MGM New Bombay Hospital, Navi Mumbai, India;; ^14^ National Institute of Cholera and Enteric Diseases, Indian Council of Medical Research, Kolkata, India;; ^15^ World Health Organization South-East Asia Regional Office, New Delhi, India

## Abstract

Typbar-TCV^®^, a typhoid conjugate vaccine (TCV), was prequalified by the World Health Organization in 2017. We evaluated its effectiveness in a mass vaccination program targeting children 9 months to 14 years in Navi Mumbai, India, from September 2018 to July 2020. We compared laboratory-confirmed typhoid cases from six clinical sites with age-matched community controls. Of 38 cases, three (8.6%) received TCV through the campaign, compared with 53 (37%) of 140 controls. The adjusted odds ratio of typhoid fever among vaccinated children was 0.16 (95% CI: 0.05–0.55), equivalent to a vaccine effectiveness of 83.7% (95% CI: 45.0–95.3). Vaccine effectiveness of Typbar-TCV in this large public sector vaccine introduction was similar to prior randomized controlled trials, providing reassurance to policymakers that TCV effectiveness is robust in a large-scale implementation.

## INTRODUCTION

*Salmonella enterica* subspecies *enterica* serovar Typhi (*S.* Typhi), the causative agent of typhoid fever, caused an estimated 11 million infections and 116,800 deaths worldwide in 2017, with children experiencing the greatest burden of disease.^1^ Typhoid is fecal-orally transmitted and South Asia and sub-Saharan Africa have the highest global incidence.^2^ Alongside typhoid vaccination, the WHO recommends water, sanitation, and hygiene (WASH) interventions, but these require longer term investment and government implementation to accomplish.^3^^–^^5^ The emergence of multidrug-resistant and extensively drug-resistant lineages have increased the need for more immediate preventive measures.^6^

Typhoid vaccines have existed for decades, including an oral live attenuated vaccine, Ty21a, and an injectable Vi capsular polysaccharide (ViPS) vaccine, but they are not licensed for children <2 years old, the Ty21a vaccine requires multiple doses, and the ViPS vaccine requires revaccination every 3 years.^3^ Typhoid conjugate vaccines (TCVs), two of which the WHO has prequalified, are highly immunogenic, can be administered to infants, and can potentially offer a longer duration of protection (estimated >7 years).^3^^,^^7^ One of these, Typbar-TCV^®^ (Bharat Biotech International Limited, Hyderabad, India), is a single-dose TCV containing the *S*. Typhi Vi capsular polysaccharide antigen conjugated to tetanus toxoid carrier protein.^8^ Multiple randomized controlled trials in endemic regions have found this vaccine to have substantial vaccine efficacy, including in Nepal (79.0%, 95% CI: 61.9–88.5), Bangladesh (85.0%, 97.5% CI: 76.0–91.0), and Malawi (80.7%, 95% CI: 64.2–89.6).^9^^–^^11^

Navi Mumbai is an urban municipality administered by the Navi Mumbai Municipal Corporation (NMMC), known to have a high burden of typhoid among children, including multidrug-resistant strains.^12^^,^^13^ Health services in NMMC are provided through 22 urban health posts that each serve defined sections of the city. In 2018, the NMMC planned the first phase of a pediatric vaccination campaign using Typbar-TCV^®^, ^14^ and approximately 113,420 children between 9 months and 14 years old received TCV.^14^ A second round of vaccination was planned for 2020 but was delayed indefinitely due to the COVID-19 pandemic. The study design included a population-based community health survey to monitor for cases and to gather participant demographic and healthcare utilization measurements. The survey included information on demographic and socioeconomic characteristics, healthcare use and hospitalization information, receipt of typhoid vaccine, history of other childhood immunizations, household wealth, and potential risk factors for typhoid. Study staff requested vaccination cards from participants who reported typhoid vaccine receipt. All data were collected using CommCare on password-protected tablets. The survey was initiated after the vaccination campaign and was ongoing between September 2018 and March 2020.^15^

To gain a better understanding of the field vaccine effectiveness of Typbar-TCV, we performed a case–control analysis in vaccine-eligible children after the first vaccination campaign phase. Cases were NMMC residents between 9 months to 14 years of age at the start of the vaccination campaign (July 2018) who received a positive *S*. Typhi blood culture between September 2018 and March 2020 at one of six surveillance sites.^14^^,^^15^ Cases were each matched up to four controls (matchit function from R “MatchIt” package, R version 4.0.4), who were enrolled from the population-based community assessment survey. Controls were considered eligible if they 1) had not experienced fever within 30 days and 2) were between 9 months and 14 years old on July 1, 2018. Controls were matched to cases by 1) date of survey (within 14 days from case enrollment) and 2) age at time of TCV vaccination (within 12 months of case). Study staff collected data from both cases and controls using a structured questionnaire administered to a parent/guardian (embedded within the population-based community health survey). Case surveys were performed after a positive blood culture was confirmed.

A child was defined as vaccinated if a respondent presented a TCV campaign vaccination card or recalled that the child received a TCV vaccine during the vaccination campaign (“TCV Card or Campaign Recall”). We estimated sample sizes based on vaccine effectiveness of 85% (i.e., odds ratio = 0.15).^9^^–^^11^ Assuming an average of three controls per case, for a range of laboratory-confirmed cases and at different levels of TCV coverage among controls (40%, 50%, 60%, 70%, and 80%) we assumed we could estimate effectiveness with reasonable precision (95% CI band of less than ± 20% effectiveness 0.20).^16^

We used conditional logistic regression to calculate the odds ratio of TCV vaccination between matched cases and controls (clogit function from R “survival” package, R version 4.0.4). We additionally conducted a subanalysis using a more lenient definition of vaccinated by including those who recalled being vaccinated with TCV at any point in time (“TCV Card or recall at any time”). We excluded cases and potential controls who refused or reported not knowing the answer to the vaccination question. We also conducted stratified analyses by age (under 5 years and 5–14 years at the time of campaign). We attempted an age-stratified analysis for those under 2 years, but the model did not converge due to the limited sample size. All code used for the analysis can be found on Github (https://github.com/chrisleboa/india_typhoid/tree/master/Vaccine%20Case%20Control%20Analysis). Because typhoid was rare in this population during the surveillance period, we assumed that the odds ratio was a reasonable estimate of the relative risk.^17^ We estimated vaccine effectiveness using the formula: (1 – conditional odds ratio of cases being vaccinated) × 100.^18^

We obtained parental/guardian written informed consent for all study participants. The evaluation protocol was approved by the Institutional Ethics Committee, Indian Council of Medical Research—National Institute of Cholera and Enteric Diseases (No. A-1/2020-IEC); WHO Research Ethics Review Committee (ERC.0002923); Centers for Disease Control and Prevention Institutional Review Board (#7026); Stanford University Institutional Review Board (IRB-39627); and institutional review committees of all evaluation sites.

**Figure 1.**
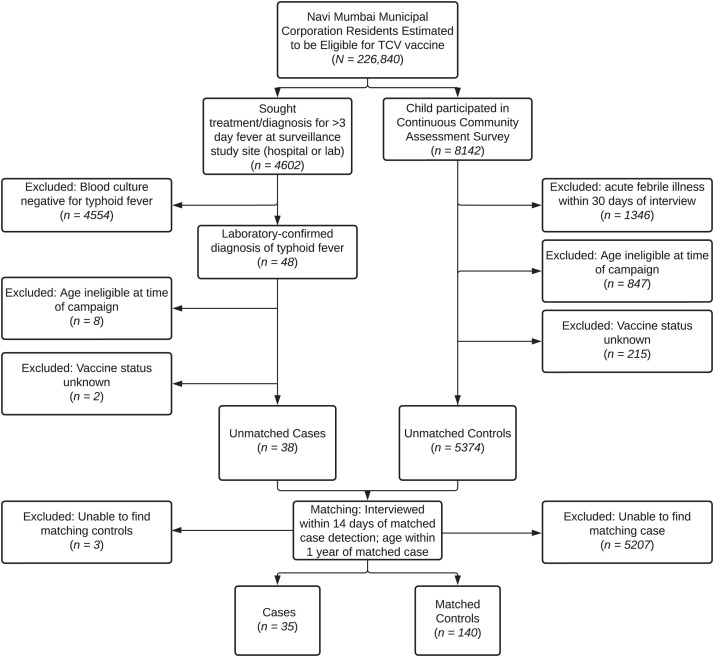
Flow chart of study design. TCV = typhoid conjugate vaccine.

A total of 38 participants had blood culture–confirmed typhoid and documented TCV vaccination status according to our primary definition. Thirty-five cases were age- and enrollment time–matched to 140 corresponding controls during the study period (September 2018–March 2020) (Figure 1). Cases and matched controls had similar demographic characteristics and household WASH components (Table 1). Most participants reported improved sanitation facilities, including flush toilets in their home, and an improved water source (Table 1).

**Table 1:** Demographic characteristics of respondent/head of household, eligible child, and household water source and sanitation information

Variable	Matched Cases (*N* = 35)	Controls (*N* = 140)	*P*-Value
Demographic characteristics of respondent or head of household			
Female sex (*n*, %)	28 (80)	111 (79)	1.00
Age 30 − 39 years (*n*, %)	22 (63)	71 (51)	0.27
Nonprofessional occupation (*n*, %)*	34 (96)	130 (93)	0.66
Middle school or lower education (*n*, %)^†^	25 (71)	71 (51)	0.04
Demographic characteristics of eligible child			
Female sex (*n*, %)	12 (34)	69 (49)	0.16
Age under 5 years (*n*, %)	8 (23)	33 (23)	1.00
Household characteristics			
Three- to four-person household (*n*, %)	20 (57)	72 (51)	0.68
Improved water source (*n*, %)^‡^	35 (100)	139 (99)	1.00
Flush toilet (*n*, %)	29 (83)	122 (87)	0.81
No. who share toilet with people outside their family (*n*, %)	5 (14)	17 (12)	0.99
Handwashing facility (*n*, %)^§^	35 (100)	131 (94)	0.30
Electricity in home (*n*, %)	35 (100)	139 (99)	1.00

* Nonprofessional occupation includes unskilled workers, trade workers, and skilled workers.

^†^ Middle school or lower education includes no formal education through Grade 10.

^‡^ Improved water source is defined as piped into dwelling, yard, or plot; public tap or standpipe; tube well or borehole; protected well; or protected spring.

^§^ Handwashing facility is defined as a place in the home or right outside the home where one is able to wash hands.

Of participants with laboratory-confirmed typhoid, 8.6% (3/35) received Typbar TCV during the campaign, compared with 38.0% (53/176) of control participants (Table 2). The conditional odds ratio of being vaccinated in the case group versus the control group was 0.16 (95% CI: 0.05–0.55). The estimated vaccine effectiveness was 83.7% (95% CI: 45.0–95.3). Our analysis of the alternative vaccination definition (expanding recall outside of campaign) decreased the estimated effectiveness of the vaccine (Table 2).

**Table 2:** TCV vaccination rates among cases and controls, and modeled estimates of vaccine effectiveness by vaccinated definition and age

Vaccinated Definition or Age	Typhoid Cases	No. of Typhoid Cases Vaccinated (%)	Matched Controls	No. of Typhoid Controls Vaccinated (%)	Modeled Vaccine Effectiveness (95% CI)
TCV card or campaign recall	35	3 (8.6)	140	53 (38.0)	83.7% (45.0–95.3)
TCV card or recall at any time	34	7 (20.0)	136	58 (43.0)	62.8% (10.7–84.5)
Age <5 years	8	1 (12.5)	33	11 (33.0)	72.3% (–144.0 to 96.9)
Age 5–14 years	27	2 (7.4)	107	42 (39.0)	87.0% (42.9–97.1)

TCV = typhoid conjugate vaccine.

We found a numerically lower effectiveness among those under 5 years (72.3%, 95% CI: –144.0–96.9) compared with children 5 to 14 years of age (87.0%, 95% CI: 42.9–97.1) (Table 2), but CIs were wide, and we were not powered to compare effectiveness between age groups. However, these findings mirror age-based TCV efficacy published in Malawi, in which efficacy was 74.4% (95% CI: 31.7–90.4) in children under 5 years at the time of vaccination compared with 83.7% (95% CI: 63.6–92.7) in older children.^11^ We did not enroll enough children under 2 years with typhoid to estimate the impact of TCV vaccination amongst this age group, but work from a phase 3 trial in Bangladesh suggests that TCV is highly protective among children under 2 years (81.0%, 95% CI: 39.0–94.0).^10^

The vaccine effectiveness described in this study (83.7%) is similar to the efficacy demonstrated in recent TCV clinical trials, which range from 80% to 85%.^9^^–^^11^ Our study did find three blood culture–confirmed cases of typhoid among TCV-vaccinated individuals. Of note, other TCV randomized controlled trials experienced typhoid infection in vaccine arms.^9^^,^^10^ The continued incidence of typhoid among those vaccinated suggests a need for additional measures, such as water and sanitation infrastructure, to potentially eliminate typhoid in endemic areas.^5^^,^^15^

This study was limited by self-report of vaccination status, which may have led to recall bias, potentially underestimating or overestimating the true proportion of vaccinated individuals; of note, Typbar-TCV has been available in India for private sector purchase since 2013.^19^ Additionally, the COVID-19 pandemic altered healthcare-seeking behavior and cut short our planned data collection activities. We anticipated enrolling more cases and controls, but enrollment was short of expectations, which may be due to the low sensitivity of even optimally collected blood cultures for typhoid fever diagnosis.^20^ More research is needed to understand who was reached and missed in mass vaccination campaigns like this one and to assess the long-term impact of TCV vaccination on population-level incidence of typhoid.

This field effectiveness evaluation of Typbar-TCV provides critical data needed for local, national, and global TCV decision-making and development of policies to reduce the burden of typhoid. Typbar-TCV had comparable vaccine effectiveness in Navi Mumbai as in other trials, and the programmatic effect of this public-sector campaign was consistent with the estimated vaccine coverage.^15^ These results provide reassuring evidence that vaccine introduction is scalable and should enable increased availability and access to TCVs in typhoid-endemic countries.
